# The effect of encoding task on the forgetting of object gist and details

**DOI:** 10.1371/journal.pone.0255474

**Published:** 2021-09-22

**Authors:** Zhongyu Hu, Wenxi Zhou, Jiongjiong Yang

**Affiliations:** School of Psychological and Cognitive Sciences, Beijing Key Laboratory of Behavior and Mental Health, Peking University, Beijing, China; University of Glasgow, UNITED KINGDOM

## Abstract

One important feature of episodic memory is that it contains fine-grained and vividly recollected details. How to improve and maintain detailed information over time has been one of the central issues in memory research. Previous studies have inconsistent findings on whether detailed memory is forgotten more rapidly than gist memory. In this study, we investigated to what extent different encoding tasks modulated forgetting of gist and detailed information. In three experiments, participants were presented pictures of common objects and were asked to name them (Experiment 1), describe the details about them (Experiment 2) or imagine scenes associated with them (Experiment 3). After intervals of 10 minutes, one day, one week and one month, gist and detailed memories of the pictures were tested and assessed using a remember/know/guess judgement. The results showed that after the naming task, gist and detailed memories were forgotten at a similar rate, but after the description and the imagination tasks, detailed memory was forgotten at a slower rate than gist memory. The forgetting rate of gist memory was the slowest after the naming task, while that of detailed memory was the slowest after the description task. In addition, when three experiments were compared, the naming task enhanced the contributions of recollection and familiarity for gist memory, while the description task enhanced the contribution of familiarity for detailed memory. These results reveal the importance of the encoding task in the forgetting of gist and detailed information, and suggest a possible way to maintain perceptual details of objects at longer intervals.

## Introduction

One important feature of episodic memory is that it contains fine-grained and vividly recollected details. By this we acquire distinct representations of similar events and retain unique experiences. For a single object, details refer to perceptual features of an object, such as its color, size and orientation [1, 2]. Correspondingly, gist refers to the concept or category attributes of an object [3–8]. In some studies, the terms of conceptual/central and perceptual recognition are also used to refer to gist and detailed memories, especially when object pictures are adopted as materials [9–11].

Gist and detailed memories are assessed by different methods in lab-based studies. When film clips or stories are recalled, the responses are coded as story-based/central themes and perceptual details separately [1, 2, 6, 12]. When objects [13] or scenes [14] are tested, one method to assess gist and detailed memories is to measure the ability to discriminate between targets and lures with different degrees of similarity [15–17]. Another method is to use word-based and picture-based tests to examine semantic/conceptual and detailed/perceptual features of the objects separately [9–11]. The latter method is based on the dual coding theory (DCT) [18] and fuzzy trace theory (FTT) [19–21], which propose that memory representations of an object are established in parallel and stored separately in two different forms, namely conceptual and perceptual representations. In addition, gist and detailed memories are retrieved independently [20, 21] and may be tested by words and pictures [7, 9–11, 22–24]. For example, in a study of Davis et al. (2021), after participants learned object images, they were presented with word labels to test their conceptual memory and presented with object pictures to test their perceptual memory [11]. Bahrick & Boucher (1968) showed that the recall performance of object names was not correlated with that of picture recognition of the same objects by the same participants [9]. It suggests that the conceptual and perceptual representations are independently stored and retrieved [9] with the passage of time [10].

Many studies have suggested that detailed memory is more subject to decay than gist memory, leading to impoverished memory for details over time, whereas gist information is relatively better retained [6, 15, 25–28]. For example, Bartlett (1932) found that with the passage of time, people retained the main contents of stories they had read, whereas their memory of detailed information was significantly decreased [25]. Similarly, in a study of Sekeres et al. (2016), participants watched a series of film clips and were asked to recall central information and perceptual details about the clips at delays of 10 minutes, three days and seven days. The results showed that memory of details decreased significantly from 10 min to 3-day and 7-day intervals, whereas that of central/gist information remained relatively stable over time [6].

Nevertheless, other studies have found that people can retain a mass of detailed memories of objects [15] for a long time [13, 29]. For example, in a study of Andermane and Bowers (2015), participants were presented with a series of pictures of common objects and decided whether the pictures were repeated or not. After 10-minute or 1-week interval, they were presented with old and lure pictures and completed a forced-choice task and a yes-no recognition task [13]. During both tasks, the lures were similar or different objects, which were used to detect detailed memory (i.e., the ability to distinguish old and similar objects) and gist memory (i.e., the ability to distinguish old and different objects). The results showed that although gist memory performance was higher than that of detailed memory, the two types of memory decreased at a similar rate over a week [26, 27]. Similarly, another study found similar forgetting rates of gist and detailed memories of objects over 3 months [30]. These results suggest that although gist information is generally recalled better than details, at least in some cases, details are not necessarily forgotten more rapidly than gist.

Clarifying the forgetting rates of detailed and gist memories is important to understand how different types of memory representations change over time. One way to reconcile the conflicting results is to consider the encoding priority of gist and detailed information and the effect of encoding task on subsequent memory. As proposed by the FTT [19–21], gist and detailed memories are encoded in parallel, stored and retrieved separately with different representations. Therefore, when gist or detailed information is emphasized during encoding, separate memory traces should be enhanced, leading to slower forgetting of specific traces over time. In addition, as gist information is usually vital to an event, adults tend to adopt the strategy of fuzzy processing to deal with central/gist information [20]. For example, film clips have a strong narrative nature. Therefore, during encoding and retrieving of film clips, participants prefer to process the central theme of the clips, which leads to a slower forgetting rate of the central information than that of the perceptual details in previous studies [6]. On the other hand, when perceptual features are emphasized during encoding, detailed information can be encoded more deliberately and maintained for a long time [13, 15]. Although these results suggest that encoding tasks modulate gist and detailed memory performance, to further clarify their forgetting patterns, the same materials and the same tests should be used for different encoding conditions [31]. To date, few such studies, if any, have been reported.

In addition, in the study of memory forgetting, one question that should not be ignored is whether the forgetting of gist and detailed memories is associated with different underlying cognitive mechanisms. Based on the dual-process model, memory recognition depends on the processes of recollection and familiarity. The former refers to the recollection of a learned item and its spatiotemporal context, and the latter refers to a sense of knowing without the ability to recall more details [32–34]. Studies have also shown that over time the contribution of recollection decreases significantly, while that of familiarity process decreases less [35, 36], remains unchanged [37, 38], and even increases [39]. Therefore, memories dependent on recollection are more likely to decline over time, while those dependent on familiarity are comparatively less likely to decline [40]. In the case that recollection and familiarity processes contribute differently to gist and detailed memories, their forgetting rates should differ. The familiarity-based memory should be forgotten at a slower rate. Furthermore, if an encoding task enhances the familiarity contribution to subsequent memory, the forgetting rate of this type of memory should become slower. In this study, this hypothesis was tested by measuring the recollection and familiarity contributions for gist and detailed memories, given three different encoding tasks.

In sum, the main objectives of this study were (1) clarify to what extent the encoding task influences the forgetting of gist and detailed memories and (2) examine how different recognition processes (recollection versus familiarity) contribute to these two types of object memory over time. Participants were presented with pictures of common objects and performed different encoding tasks. In Experiment 1, participants were asked to name the objects [11]. During this naming task, they were oriented to pay more attention to gist/conceptual information. In Experiment 2, participants were asked to describe perceptual features of the objects [41, 42]. During this description task, they were oriented to process detailed/perceptual information. In Experiment 3, participants were asked to imagine scenes associated with the objects [43, 44]. During this imagination task, neither conceptual nor perceptual information was emphasized. After 10 minutes, 1 day, 1 week and 1 month, the participants’ gist and detailed memories were tested separately by two different recognition tasks. Recognition differs from recall tasks in that a cue is provided to diminish the possibility of inaccessibility [45]. To test gist memory, old and new names (e.g., schoolbag, apple) of the objects were presented and the participants made an old/new name judgment [7, 11, 22]. To test detailed memory, the old and similar pictures of the objects were presented and the participants made an old/new object judgment. After making old/new judgments on the gist and details of the objects, the participants were asked to make remember/know/guess judgments (RKG) [34] to dissociate the contribution of recollection and familiarity processes.

Most previous studies focused on the characteristics of forgetting within one week [13, 14]. To obtain forgetting patterns of more remote intervals, a time point of 1 month was additionally included. To control for initial memory performance, the forgetting rate for each memory type was calculated as the proportion of the decrease in memory accuracy relative to the accuracy at 10 minutes after encoding. According to the FTT [19–21] and previous research [6, 12–14, 41], we predicted that as the naming task emphasizes gist information, the gist memory should be higher and its forgetting should be slower after the naming task than after the description and the imagination tasks. In contrast, as the description task emphasizes detailed information, detailed memory should be enhanced and should be retained for a longer time after the description task than after the naming and the imagination tasks.

## Experiment 1

### Materials and methods

#### Participants

Twenty-six healthy college students (12 females, age = 20.38 ± 2.12 years) participated in the experiment. The overall sample size for the experiment was based on an a prior power analysis (G*Power 3.1.9.6; University of Kiel, Germany). In order to obtain adequate power (i.e., alpha value = 0.05, 1−β = 0.95) and detect a moderate effect size (i.e., f = 0.25) for the interaction of retention interval (4) and memory type (2), we would need a total sample of at least 23 participants for each experiment. Note that as a between-subjects design was used, the effect size we chose was at a medium level to obtain more credible results. All the participants were right-handed, with normal or corrected-to-normal vision. They all provided written informed consent in accordance with the procedures and protocols approved by the Review Board of School of Psychological and Cognitive Science, Peking University.

#### Stimuli

Two factors were included in Experiment 1, with memory type (gist, detail) and retention interval (10 min, 1 day, 1 week, 1 month) as within-subjects factors.

Two hundred and forty pairs of color pictures of objects were selected from Hemera (http://www.hemera.com/hemera) and the Internet (http://www.baidu.com), which represented 240 distinct concepts (e.g., clip, kiwi). Among them, 68 were natural objects, while 172 were man-made objects. Each object exemplar had two versions (Detail A and Detail B), such that each version contained different details, such as the orientation, number of elements involved, and the color of the object.

The material selection was based on the evaluation results obtained in another group of participants (n = 23, 13 females, age = 22.43 ± 2.27 years). The evaluation included picture naming, picture familiarity and picture similarity. During the familiarity rating, participants judged to what extent they were familiar with the presented picture (1- very familiar, 5- very unfamiliar). During the similarity rating, they judged to what extent two pictures of the same concept were similar (1- very similar, 5- completely different). The selected pictures had high naming accuracy (0.94 ± 0.07), high familiarity (1.78 ± 0.27) and moderate degree of similarity (2.93 ± 0.35). Based on the naming accuracy, the corresponding concept words for gist memory test were selected (number of characters: 2.20 ± 0.60, number of strokes: 18.05 ± 7.31; logarithmic word frequency (17.81 ± 1.37) [46].

All of the pictures (240 different concepts, 480 pictures) were divided into four groups. The four groups were randomly assigned to four conditions (i.e., old and new concept words for gist memory test; old and new/similar pictures for detailed memory test). During encoding, only the pictures of three groups were randomly presented, and for each pair, only one picture was randomly learned. Each group was further randomly divided into four sets for different retention intervals. Finally, at each interval, 15 old concepts and 15 new concepts were used for gist memory test, and 15 old pictures and 15 new/similar pictures were used for detailed memory test. The materials used for the memory tests were all different, but were counterbalanced among the participants, so that different pictures had equal chance to be used as materials under each condition.

#### Procedure

**Fig 1:**
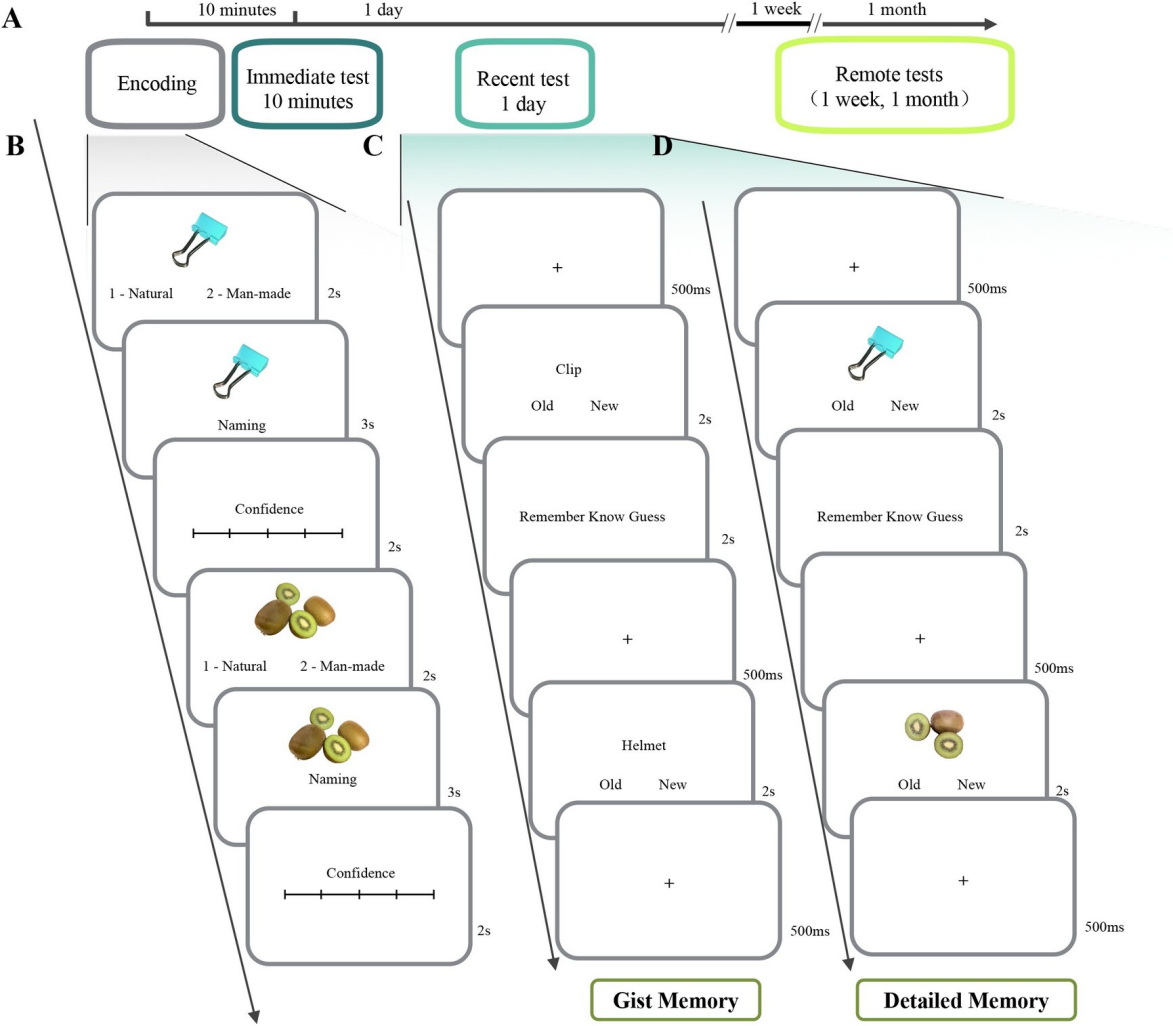
Experimental procedure in Experiment 1. The participants learned all the pictures on the same day, and performed the two recognition tasks at four different intervals **(A)**. During the encoding phase, participants were presented with each of the pictures, and judged whether the picture was natural or man-made, then named the object with confidence rating **(B)**. During the test phase, the gist memory **(C)** and detailed memory **(D)** were tested separately. The participants made old/new judgments for gist and detailed information. If the judgment was “old”, they further made a remember/know/guess judgment. The object images used in this figure are similar but not identical to the original images used in our study, and are therefore for illustrative purposes only.

The experiment included one encoding phase and four retrieval phases (Fig 1A). The participants learned 180 pictures of the objects on the same day, and performed the recognition tasks at four different intervals.

During the encoding phase (Fig 1B), to reduce individual differences in processing the pictures before encoding task manipulations, each of the pictures was presented on the center of the screen for 2 seconds and the participants were asked to determine whether the object was natural or man-made. Then, the picture was presented again for 3 seconds, and the participants were asked to name the object and rate the confidence level (1—very uncertain, 5—very certain). The participants were asked to practice nine trials to make sure that they understood the requirements of the naming and the rating tasks. Among the 180 pictures, 60 pictures belonged to the old concept set, 60 pictures belonged to the old picture set and the other 60 pictures belonged to the new/similar picture set in the subsequent memory test. The pictures were presented in a pseudorandom order, so that no more than three stimuli in the same condition were presented consecutively.

During each of the retrieval phases, gist and detailed memories were tested separately. In the gist memory test (Fig 1C), the participants were presented with each of 30 names of the objects (e.g., “dog” or “truck”) for 2 s. Then they decided whether the object the name denoted had been represented during encoding. They were asked to respond as quickly and as accurately as possible. This ensured that the participants made the judgment within 2s and balanced the contribution of recollection and familiarity processes [34, 47]. Among them, 15 names were concepts of the pictures learned during the encoding phase (i.e., old concepts) and 15 had not been learned (i.e., new concepts). When the participants judged a name as an "old concept", they further made a remember/know/guess (RKG) judgment [33, 34, 48]. The RKG instruction of gist memory was described as follows: “The remember judgment implies that you could vividly recall the concept name according to previously learned object. The know judgment implies that you could recall the name, but feel that the name is familiar without any specific information about the object. If you could not retrieve the name by the two aforementioned processes, you may choose response of guess”.

In the detailed memory test (Fig 1D), the participants were presented with each of the 30 pictures for 2 s and judged as quickly and as accurately as possible whether the picture had been learned during the encoding phase. Among them, 15 pictures were the same as what they had learned (i.e., old pictures) and 15 were pictures with the same concepts but different details (i.e., similar pictures). Similarly, when the participants judged the picture as an "old picture", they further made the RKG judgment. The main RKG instruction of detailed memory was described as follows: “The remember judgment implies that you could vividly recall the object details and other contexts. The know judgment implies that you forget the details of the object and contexts, but retain an impression of seeing the image. If you could not retrieve any details and contexts of the object and did not feel familiar with it, you may choose guess”.

In the gist and detailed memory tests, the materials were presented in a pseudorandom order so that no more than three stimuli in the same condition were presented consecutively. As only the old concepts were included in the detailed memory test, to diminish the influence of two memory tests, the gist memory test was performed before the detailed memory test. The button pressed for the recognition judgment was counterbalanced across the participants.

Before the test phase at 10-min interval, to avoid a rehearsal from the encoding phase, the participants were asked to count backward by 7 continuously from 1000 for 5 minutes as a distractor task. The participants had separate opportunities to practice test trials before the formal phases. In particular, to ensure that they followed the instruction of the RKG procedure, they specifically practiced this part with feedback from experimenters. The participants were not aware what they would be tested until the first test practice, so that knowledge of the kinds of memory tests administered (i.e., detailed and gist-based) would not affect the encoding process. Additionally, the participants were asked to not deliberately rehearse the stimuli among different retention intervals.

#### Data analysis

The corrected recognition accuracy (Hit rate—FA rate) and reaction times (RTs) were analyzed using a repeated-measures analysis of variance (ANOVA), with retention interval (10 min, 1 day, 1 week, 1 month) and memory type (gist, detail) as within-subjects factors. As the results of the corrected recognition and d’ values were similar, only the former one was reported. The results of the hit rate and false alarm (FA) rate were presented in S1 Table. The forgetting rate was estimated by the interaction between the time interval and memory type [49–51]. The normality and sphericity for each analysis (including *t*-tests and ANOVAs) were checked and all of analyses met the assumptions. Partial Eta Squared (η^2^) was calculated to estimate the effect size of each analysis. Post-hoc pairwise comparisons were Bonferroni-corrected (*p* < 0.05, two-tailed). All the results of the post-hoc comparisons related to retention interval were listed in S2 and S3 Tables.

To exclude the influence of initial memory performance (i.e., accuracy at 10-min), the forgetting rates (Fr) were calculated for each participant as follows and analyzed by t-test for the two memory tasks:

$$Fr=\frac{accuracy\;at\;10\;minutes–accuracy\;at\;1\;month}{accuracy\;at\;10\;minutes}$$

In addition, to better illustrate the memory change over time, the forgetting curves were fitted [52, 53] to estimate the forgetting pattern with the group data of each task. The forgetting curve is a power function curve decided by the initial memory performance (β), forgetting rate (ψ) and test time (t):

$$accuracy=\beta \times t^{-\psi }$$

The recollection and familiarity processes were estimated by an independent “remembering-knowing” procedure [34, 54] and were corrected according to the FA rate [55, 56]. The recollection process was estimated using the following equation:

Recollection=p(R,Hit)–p(R,FA)

while the familiarity process was estimated as follows:

$$Familiarity=\frac{p(K,Hit)}{1-p(R,Hit)}-\frac{p(K,FA)}{1-p(R,FA)}$$

The ‘p (R, Hit)’ implies the proportion of the recollected hits to all the old items, and ‘p (R, FA)’ implies the proportion of the recollected false alarms to all the new items. By this, the R and K responses are not only mutually exclusive, but also independently estimated. Repeated measures ANOVAs were applied separately for the recollection and familiarity processes, with memory type and retention interval as within-subjects factors (*p* < 0.05, two-tailed).

#### Results of Experiment 1

*Encoding results*. During encoding, the participants showed high accuracy of natural/artificial judgment (0.91 ± 0.15), high naming accuracy (0.96 ± 0.02) and high naming confidence (4.75 ± 0.19). There were no significant effects of memory type, retention interval and their interaction for the natural/artificial judgement, naming performance and confidence level (*p’* s > 0.30).

**Fig 2:**
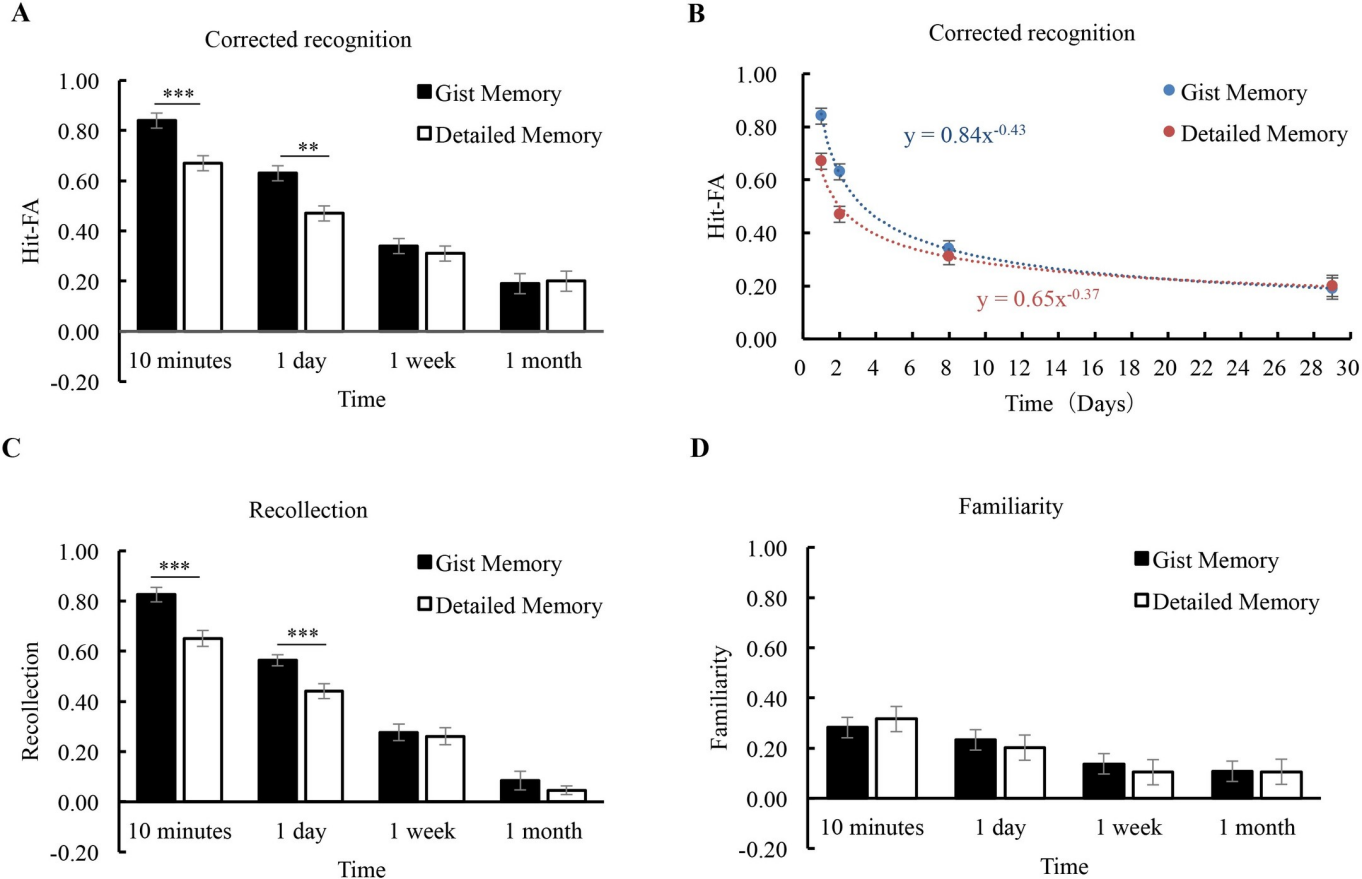
Results of Experiment 1. The corrected recognition was shown for each condition **(A)**. Forgetting curves were fitted to estimate the forgetting rates for gist and detailed memories **(B)**. Estimates of the contributions of recollection and familiarity were shown for each condition **(C, D)**. The error bars represent the standard errors of the means. * *p* < 0.05; ** *p* < 0.01; *** *p* < 0.001.

*Corrected recognition*. The ANOVA for Hit–FA showed a significant effect of retention interval (*F* (3, 75) = 104.75, *p* < 0.001, *η*^*2*^ = 0.81) (Fig 2A). Memory performance declined significantly over time (*p’*s < 0.05). The main effect of memory types was significant (*F* (1, 25) = 16.19, *p* < 0.001, *η*^*2*^ = 0.39), showing that gist memory was higher than detailed memory. The interaction between memory type and retention interval was also significant (*F* (3, 75) = 5.63, *p* = 0.004, *η*^*2*^ = 0.18). Further analysis showed that at 10 minutes and 1 day, the performance of gist memory was significantly higher than that of detailed memory (*p’*s < 0.001), but at 1 week and 1 month, there was no significant difference between gist and detailed memories (*p’*s > 0.30). These results suggest that after the naming task, gist information is better retained than that of detailed information at shorter intervals, but this advantage disappears as gist memory declined rapidly from 1 day to 1 week.

*RTs*. The ANOVA showed that the RTs of gist memory (1.07 s ± 0.12 s) were significantly shorter than that of detailed memory (1.11 s ± 0.11 s) (*F* (1, 25) = 4.97, *p* = 0.035, *η*^*2*^ = 0.17). The interaction between memory type and retention interval was significant (*F* (3, 75) = 6.13, *p* = 0.001, *η*^*2*^ = 0.20). Further analysis showed that at 10 minutes, the RTs of gist memory were significantly shorter than those of detailed memory (*p* < 0.001), but at later retention intervals, the RTs were comparable for the two memory tests (S1A Fig).

*Forgetting rate*. When the initial performance at 10-min was controlled, the results showed that there was no significant difference between the forgetting rates (Fr) of gist memory (0.76 ± 0.23) and detailed memory (0.71 ± 0.29) (*t* (25) = 0.67, *p* = 0.51, *Cohen’s d* = 0.13). Curves for the group were fitted for illustration purposes (Fig 2B). Gist memory declined in line with the curve *m*_gist_ *=* 0.84* t ^-0.43^ (*R*^*2*^ = 0.69, *p* < 0.001). Similarly, the trend of detailed memory was well fitted by the curve *m*_detail_ = 0.65* *t* ^-0.37^ (*R*^*2*^ = 0.51, *p* < 0.001). These results suggest that the gist and detailed memories decline at a similar rate after the naming task.

*Recollection and familiarity*. The contribution of recollection declined rapidly from 10 minutes to 1 month (*F* (3, 75) = 167.75, *p* < 0.001, *η*^*2*^ = 0.87) (Fig 2C) and was higher for gist memory than for detailed memory (*F* (1, 25) = 16.52, *p* < 0.001, *η*^*2*^ = 0.40). There was a significant interaction between memory type and retention interval (*F* (3, 75) = 5.36, *p* = 0.002, *η*^*2*^ = 0.18). Further analysis showed that at 10 minutes and 1 day, the recollection contribution was higher for gist memory than for detailed memory (*p*’s < 0.001), but at 1-week and 1-month, there was no significant difference between the two memory types (*p*’s > 0.30). For the contribution of familiarity, it decreased significantly over time (*F* (3, 75) = 10.15, *p* < 0.001, *η*^*2*^ = 0.29) (Fig 2D). The main effect of memory type (*F* (1, 25) = 0.04, *p* = 0.85, *η*^*2*^ = 0) and the interaction between memory type and retention interval (*F* (3, 75) = 0.23, *p* = 0.77, *η*^*2*^ = 0.01) were not significant. In addition, the contributions of recollection and familiarity for each condition were higher than the chance level (*p*’s < 0.05). The results suggest that the recollection process contributes more to gist memory than detailed memory, especially at shorter intervals, but the contribution of familiarity is comparable for both gist and detailed memories.

In this experiment, the verbal reports of the concepts were consciously generated during encoding, which could enhance the extraction of gist from visual stimuli (e.g., pictures) and thus improve the accuracy of gist memory. Also note that gist and detailed memories were tested in different test formats [7, 9–11]. This may confound the results; we discuss this limitation in detail in the Discussion section.

In conclusion, the results of Experiment 1 showed that after encoding objects by naming them, gist memory was stronger than detailed memory at the two shorter intervals, which were more associated with a contribution of recollection. However, the two types of memory (i.e., gist and details) had similar forgetting rates over time. In Experiment 2, we adopted another encoding task (i.e., description) that emphasized processing of detailed information to explore its effect on memory and the forgetting of gist and detailed information.

## Experiment 2

### Materials and methods

#### Participants

Twenty-six college students participated in this experiment (9 females, mean age = 21.77 ± 2.70 years). The sample size was determined by the power analysis using G-power program (G*Power 3.1.9.6; University of Kiel, Germany). A prior power analysis revealed that a total sample size of at least 23 participants would provide 95% power to detect the interaction between memory type and retention interval. All the participants were right-handed, with normal or corrected-to-normal vision. They all provided written informed consent in accordance with the procedures and protocols approved by the Review Board of School of Psychological and Cognitive Science, Peking University.

#### Stimuli, procedure and data analysis

The materials used in Experiment 2 were the same as those used in Experiment 1. The procedure was the same except for the encoding task. For each trial, after the participants made a natural/man-made judgment, they were asked to mentally describe the features of the image as detailed as possible for 3 seconds, such as the orientation, number of elements involved, the shape and color of the object. Then they rated the level of detail about their descriptions (1—not detailed, 5—very detailed). Before the formal experiment, the participants sufficiently practiced nine trials and verbally reported their descriptions to ensure that they followed the instructions. For example, when an image of a life buoy was presented during practice, they may describe it as ‘round, big’ or ‘round, orange, silver striped’. The analyses of the corrected recognition, RTs, forgetting rate (Fr), contributions of recollection and familiarity in Experiment 2 were the same as those were described in Experiment 1. All of the analyses met the assumptions, except for the ‘Fr’ of detailed memory (Shapiro-Wilk test, *p* = 0.01). So we performed the next two steps to reanalyze the related data. First, the ‘Fr’ of detailed memory was normally distributed when two outliers (Fr < 2SD) were excluded (method 1, Shapiro-Wilk test, p = 0.80) or when the outliers were replaced by the expectation maximization imputation [57] (method 2, Shapiro-Wilk test, *p* = 0.93). The related analyses of *t*-test and ANOVA were performed for the two methods and the results were similar. Second, nonparametric tests for the ‘Fr’s including the two outliers were performed. The nonparametric results were the same as the ANOVA results, so the latter with method 2 was reported in the text.

#### Results of Experiment 2

*Encoding results*. During encoding, the participants showed high accuracy of natural/artificial judgement (0.89 ± 0.14) and moderate level of detail (3.50 ± 0.55). The 2 (memory type) * 4 (retention interval) ANOVAs showed no significant effects and interactions for the natural/artificial judgement and the vividness rating (*p’* s > 0.20).

**Fig 3:**
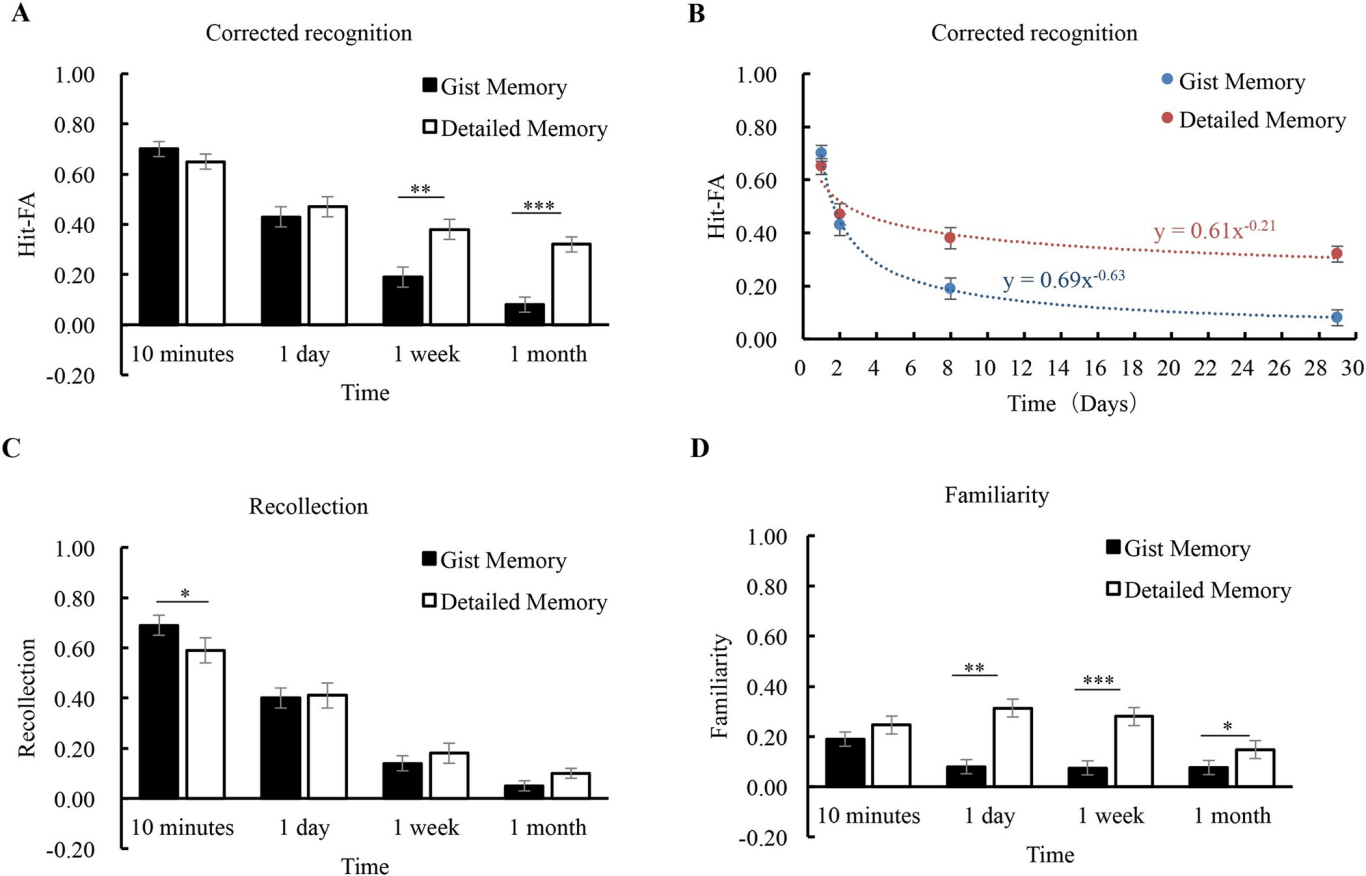
Results of Experiment 2. The corrected recognition was shown for each condition **(A)**. Forgetting curves were fitted to estimate the forgetting rates of gist and detailed memories **(B)**. Estimates of the contributions of recollection and familiarity were shown for each condition **(C, D)**. The error bars represent the standard errors of the means. * *p* < 0.05; ** *p* < 0.01; *** *p* < 0.001.

*Corrected recognition*. The corrected recognition declined significantly from 10 minutes to 1 month (*F* (3, 75) = 97.30, *p* < 0.001, *η*^*2*^ = 0.80), and the comparisons between intervals were all significant (*p*’s < 0.05). The main effect of memory type was significant (*F* (1, 25) = 19.35, *p* < 0.001, *η*^*2*^ = 0.44). Different from the results of Experiment 1, the performance of detailed memory was significantly higher than that of gist memory in Experiment 2. The interaction between memory type and retention interval was also significant (*F* (3, 75) = 9.15, *p* < 0.001, *η*^*2*^ = 0.27). Further analysis showed that unlike Experiment 1, the performance of gist memory was comparable to that of detailed memory at 10 min (*p* = 0.19) and 1 day (*p* = 0.41), while the detailed memory accuracy was higher than gist memory at 1 week and 1 month (*p*’s < 0.01) (Fig 3A).

*RTs*. The ANOVA showed that the main effect of retention interval was not significant (*F* (3, 75) = 2.22, *p* = 0.11, *η*^*2*^ = 0.08). The RTs of gist memory (1.26 s ± 0.12 s) and detailed memory (1.27 s ± 0.11 s) were similar (*F* (1, 25) = 0.29, *p* = 0.60, *η2* = 0.01). There was a significant interaction between retention interval and memory type (*F* (3, 75) = 5.21, *p* = 0.004, *η*^*2*^ = 0.17). Similar to that in Experiment 1, the faster RTs to gist (vs. detailed) information was only significant at 10-minute (*p* = 0.01). At 1 month, the RT of detailed memory was significantly shorter than that of gist memory (*p* = 0.03) (S1B Fig).

*Forgetting rate*. When the initial performance at 10 minutes was controlled, different from the results of Experiment 1, the *t*-test showed that the forgetting rate (Fr) of detailed memory (0.44 ± 0.40) was significantly slower than that of gist memory (0.90 ± 0.25) (*t* (25) = 4.64, *p* < 0.001, *Cohen’s d* = 0.91). After the distribution was corrected to meet the normality assumption, the Fr of detailed memory (0.53 ± 0.26) was significantly slower than that of gist memory (0.90 ± 0.25) (*t* (25) = 5.34, *p* < 0.001, *Cohen’s d* = 1.05). For presentation, the curves were fitted for the group (Fig 3B), which indicated that the performance of gist memory fitted the power function *m*_gist_ = 0.69 * *t* ^-0.63^ (*R*^*2*^ = 0.62, *p* < 0.001), and the performance of detailed memory fitted with the function *m*_detail_ = 0.61 * *t* ^-0.21^ (*R*^*2*^ = 0.28, *p* < 0.001).

*Recollection and familiarity*. The contribution of recollection decreased significantly over time (*F* (3, 75) = 137.40, *p* < 0.001, *η*^*2*^ = 0.85) (Fig 3C). The main effect of memory type was not significant (*F* (1, 25) = 0.001, *p* = 0.98, *η*^*2*^ = 0.001), but the interaction between memory type and retention interval was significant (*F* (3, 75) = 2.93, *p* = 0.04, *η*^*2*^ = 0.11). Further analysis showed that at 10 minutes, the recollection contributed more to gist memory than to detailed memory (*p* = 0.04), but over time, there were no significant differences between gist and detailed memories (*p*’s > 0.10). For the contribution of familiarity, it remained stable over time (*F* (3, 75) = 1.71, *p* = 0.19, *η*^*2*^ = 0.06). There was a significant effect of memory type (*F* (1, 25) = 40.81, *p* < 0.001, *η*^*2*^ = 0.62), as familiarity contributed more to detailed memory than to gist memory (Fig 3D). The interaction was significant (*F* (3, 75) = 3.07, *p* = 0.05, *η*^*2*^ = 0.11), showing that the higher contribution of familiarity for detailed memory was there from 1 day to 1 month (*p*’s < 0.05). In addition, the contributions of recollection and familiarity for each condition were significantly higher than the chance level (*p*’s < 0.01), so the results were not due to the floor effect.

In conclusion, the results of Experiment 2 showed that after the description encoding, detailed memory was stronger than gist memory at the two longer intervals. Compared to gist memory, detailed memory was more associated with the familiarity contribution, and was forgotten at a slower rate.

## Experiment 3

### Materials and methods

#### Participants

Twenty-six college students participated in this experiment (14 females, mean age = 21.92 ± 2.60 years). The sample size was determined by a power analysis using G-power program (G*Power 3.1.9.6; University of Kiel, Germany). A prior power analysis revealed that a total sample size of at least 23 participants would provide 95% power to detect the interaction between memory type and retention interval. All the participants were right-handed, with normal or corrected-to-normal vision. They all provided written informed consent in accordance with the procedures and protocols approved by the Review Board of School of Psychological and Cognitive Science, Peking University.

#### Stimuli, procedure and data analysis

The materials used in Experiment 3 were the same as those used in Experiments 1 and 2. The procedure was the same except for the encoding task. For each trial, after the participants made a natural/man-made judgment, they were asked to mentally imagine a proper scene that was associated with the object as vividly as possible for 3 seconds. Then they rated the vividness of their imagination (1—not vividly, 5—very vividly). Before the formal experiment, the participants sufficiently practiced nine trials to ensure that they followed the instructions. The analyses of the corrected recognition, RTs, forgetting rate (Fr), and the contributions of recollection and familiarity were the same as those in Experiments 1 and 2.

In order to directly compare the effects of the encoding task on gist and detailed memories, the corrected recognition, RTs, forgetting rates and the contributions of recollection and familiarity were analyzed by a repeated measures ANOVA separately, with encoding task (naming, description and imagination) as an additional between-subjects factor.

#### Results of Experiment 3

*Encoding results*. During encoding, the participants showed high accuracy of natural/artificial judgement (0.89 ± 0.16) and moderate level of imagination vividness (3.60 ± 0.51). The 2 (memory type) * 4 (retention interval) ANOVAs showed no significant effects and interactions for the natural/artificial judgement and the vividness rating (*p’* s > 0.50).

**Fig 4:**
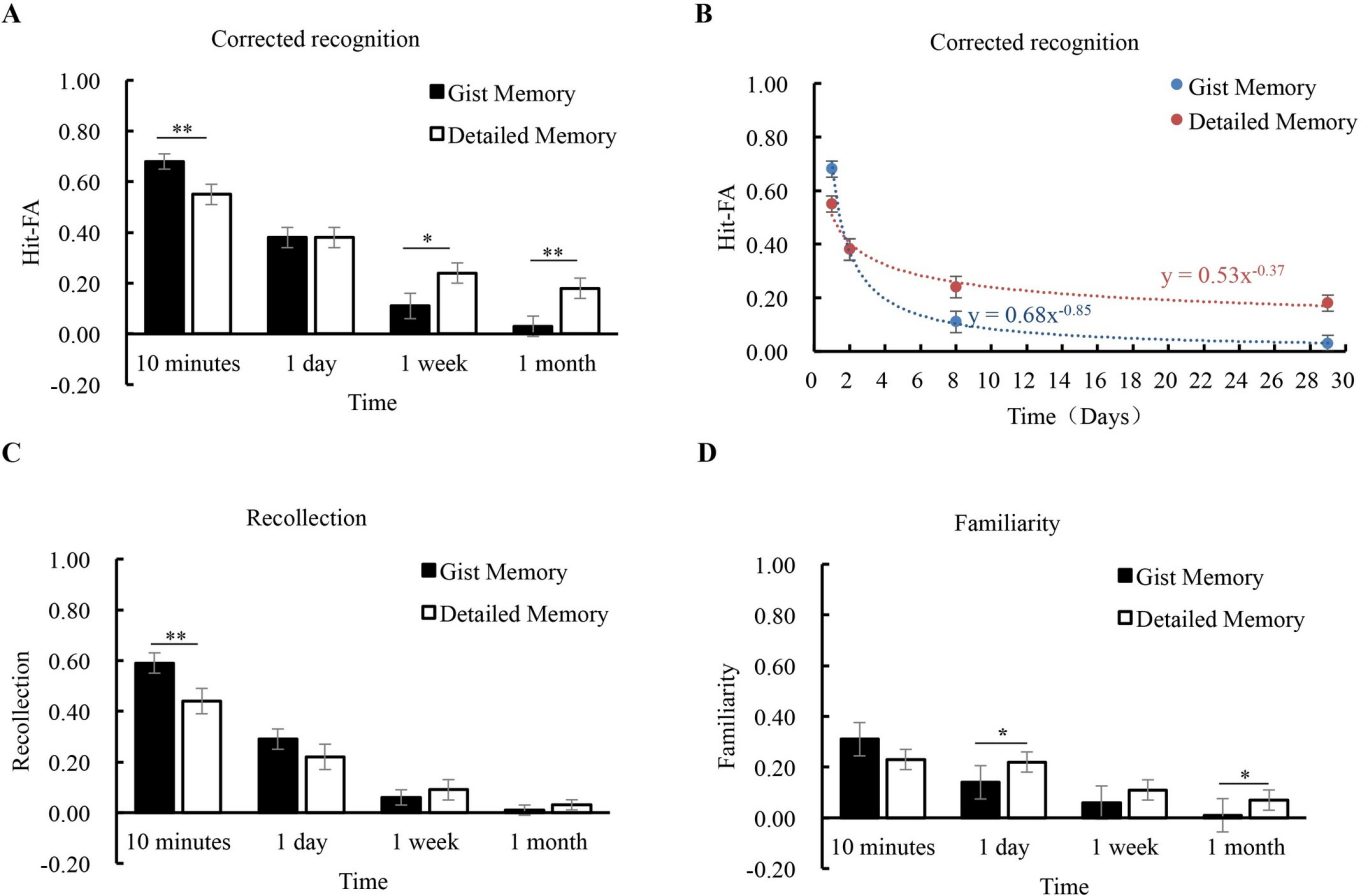
Results of Experiment 3. The corrected recognition was shown for each condition **(A)**. Forgetting curves were fitted to estimate the forgetting rates of gist and detailed memories **(B)**. Estimates of the contributions of recollection and familiarity were shown for each condition **(C, D)**. The error bars represent the standard errors of the means. * *p* < 0.05; ** *p* < 0.01; *** *p* < 0.001.

*Corrected recognition*. The corrected recognition declined significantly from 10 minutes to 1 month (*F* (3, 75) = 79.29, *p* < 0.001, *η*^*2*^ = 0.76), and the comparisons between intervals were significant (*p*’s < 0.05) except for 1 week vs. 1 month (*p* = 0.49). Different from the results of Experiment 1 and 2, the main effect of memory type was not significant (*F* (1, 25) = 2.18, *p* = 0.15, *η*^*2*^ = 0.08), showing that gist and detailed memories had comparable accuracy. The interaction between memory type and retention interval was significant (*F* (3, 75) = 6.49, *p* = 0.001, *η*^*2*^ = 0.21). Further analysis showed that the accuracy of gist memory was significantly higher than that of detailed memory at 10 minutes (*p* = 0.01), while the pattern was opposite at 1 week and 1 month (*p*’s < 0.05) (Fig 4A).

*RTs*. The main effect of retention interval (*F* (3, 75) = 2.05, *p* = 0.14, *η*^*2*^ = 0.08) and the interaction (*F* (3, 75) = 1.22, *p* = 0.31, *η*^*2*^ = 0.05) were not significant. The RTs of gist memory (1.29 s ± 0.08 s) was longer than detailed memory at a marginal level (1.26 s ± 0.11 s) (*F* (1, 25) = 3.96, *p* = 0.06, *η*^*2*^ = 0.14) (S1C Fig).

*Forgetting rate*. When the initial performance at 10 minutes was controlled, different from the results of Experiment 1, the *t*-test showed that the forgetting (Fr) of detailed memory (0.67 ± 0.39) was significantly slower than that of gist memory (0.95 ± 0.34) (*t* (25) = 2.60, *p* = 0.02, *Cohen’s d* = 0.51). After the distribution was corrected to meet the normality assumption (one outlier with Fr < 2SD was excluded), the Fr of detailed memory (0.71 ± 0.35) was significantly slower than that of gist memory (0.95 ± 0.34) (*t* (25) = 2.60, *p* = 0.02, *Cohen’s d* = 0.51). For presentation, we did the curve fitting for the group (Fig 4B). The performance of gist memory fitted the power function *m*_gist_ = 0.68 * *t* ^-0.85^ (*R*^*2*^ = 0.62, *p* < 0.001), and the performance of detailed memory fitted with the function *m*_detail_ = 0.53 * *t* ^-0.37^ (*R*^*2*^ = 0.34, *p* < 0.001).

*Recollection and familiarity*. The contribution of recollection decreased significantly over time (*F* (3, 75) = 85.18, *p* < 0.001, *η*^*2*^ = 0.77) (Fig 4C). The decline was significant among 10 minutes, 1 day and 1 week (*p*’s < 0.05) except for the interval between 1 week and 1 month (*p* = 0.15). The main effect of memory type was marginally significant (gist memory > detailed memory) (*F* (1, 25) = 3.23, *p* = 0.08, *η*^*2*^ = 0.11), and the interaction between memory type and retention interval was significant (*F* (3, 75) = 5.32, *p* = 0.002, *η*^*2*^ = 0.18). Further analysis showed that at 10 minutes, the recollection contributed more to gist memory than to detailed memory (*p* = 0.01), but over time, there were no significant differences between gist and detailed memories (*p*’s > 0.10). For the contribution of familiarity, it decreased over time (*F* (3, 75) = 12.99, *p* < 0.001, *η*^*2*^ = 0.34). There was no significant effect of memory type (*F* (1, 25) = 1.59, *p* = 0.22, *η*^*2*^ = 0.06), as the contribution of familiarity was comparable for the detailed and gist memories (Fig 4D). The interaction was marginally significant (*F* (3, 75) = 2.45, *p* = 0.07, *η*^*2*^ = 0.09), showing that the contribution of familiarity for detailed memory was higher than that for gist memory at 1 day and 1 month (*p*’s < 0.05). Note that the contributions of recollection and familiarity for each condition were higher than the chance level (*p*’s < 0.02) except for gist (*p* = 0.81) and detailed recollection (*p* = 0.17) at 1 month, and gist familiarity at 1 month (*p* = 0.71).

In conclusion, the results of Experiment 3 showed that after the imagination encoding, detailed memory was forgotten at a slower rate than gist memory. In addition, detailed memory was stronger than gist memory at the two longer intervals, and it was more associated with the familiarity contribution, especially at 1 month.

## Comparison of three experiments

**Fig 5:**
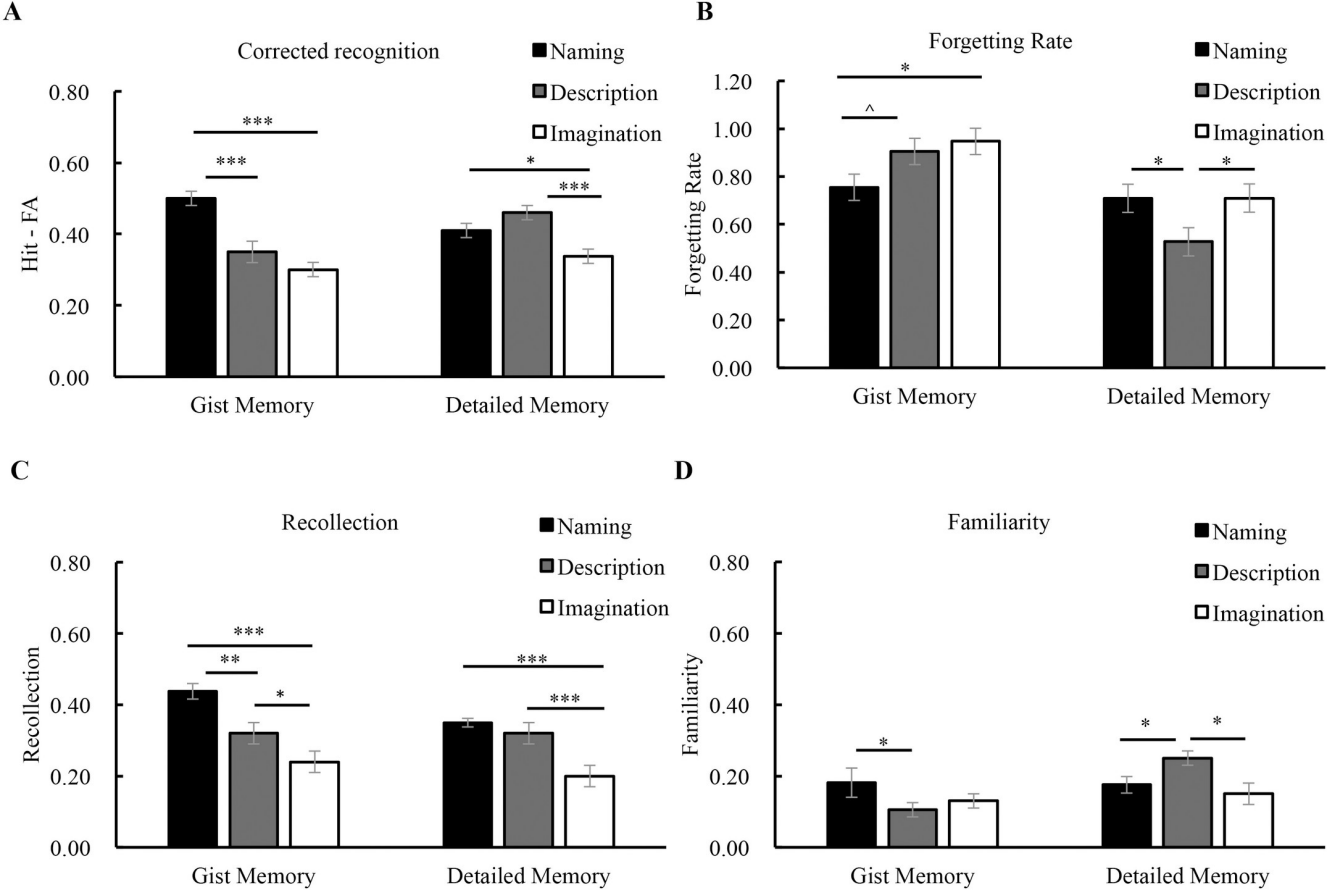
Effect of encoding task on subsequent memory and forgetting. The gist memory accuracy was the highest after the naming task, and the detailed memory accuracy was the lowest after the imagination task **(A)**. The forgetting of gist memory was the slowest after the naming task, while the forgetting of detailed memory was the slowest after the description task **(B)**. Both the recollection **(C)** and familiarity **(D)** contributed more to gist memory after the naming task than after the description and the imagination tasks. The familiarity contributed more to detailed memory after the description task **(D)**. The error bars represent the standard errors of the means. * *p* < 0.05; ** *p* < 0.01; *** *p* < 0.001; ^ *p* < 0.10.

For the corrected recognition, the result of the ANOVA (encoding task * memory type * retention interval) showed a significant interaction between memory type and encoding task (*F* (2, 75) = 17.37, *p* < 0.001, *η*^*2*^ = 0.32). The gist memory accuracy was the highest after the naming tasks (*p’s* < 0.001), while it was comparable after the description and the imagination tasks (*p* = 0.14). In contrast, the detailed memory accuracy was the lowest after the imagination task (*p’s* < 0.02), while it was comparable after the naming and the description tasks (*p* = 0.15) (Fig 5A). The 3-way interaction was not significant (*F* (6, 225) = 0.54, *p* = 0.77, *η*^*2*^ = 0.01).

For the forgetting rate (Fr) from 10-min to 1-month, there was a significant interaction between memory type and encoding task (*F* (2, 75) = 5.45, *p* = 0.006, *η*^*2*^ = 0.13). The forgetting of gist memory was the slowest after the naming task (vs. description, *p* = 0.056; vs. imagination, *p* = 0.02), while the forgetting of detailed memory was the slowest after the description task (vs. naming, *p* = 0.01; vs. imagination, *p* = 0.02). In addition, gist memory was forgotten at a similar rate after the description and the imagination tasks (*p* = 0.60), but detailed memory was forgotten more slowly after the naming task than after the imagination task (*p* = 0.02) (Fig 5B). After the distribution was corrected to meet the normality assumption, the interaction between memory type and encoding task remained significant (*F* (2, 75) = 4.62, *p* = 0.013, *η*^*2*^ = 0.11), with the lowest forgetting rate of gist memory after the naming task (vs. description, *p* = 0.056; vs. imagination, *p* = 0.02) and the lowest forgetting rate of detailed memory after the description task (vs. naming, *p* = 0.03; vs. imagination, *p* = 0.03) (Fig 5B).

For the RTs, the ANOVA showed a significant main effect of encoding task (*F* (2, 75) = 27.41, *p* < 0.001, *η*^*2*^ = 0.42), as the participants made judgment faster after the naming than after the description and the imagination tasks. Although the interaction between memory type and encoding task was significant (*F* (2, 75) = 4.62, *p* = 0.01, *η*^*2*^ = 0.11), the pattern remained the same, which indicated that the difference between encoding task did not differ for each memory type.

For the recollection process, a repeated measure ANOVA showed that its contribution was the highest after the naming task, than after the description task, with the lowest after the imagination task (*F* (2, 75) = 19.20, *p* < 0.001, *η*^*2*^ = 0.33). The recollection contributed more to gist memory than to detailed memory (*F* (1, 75) = 9.71, *p* = 0.003, *η*^*2*^ = 0.12). There was a significant interaction between encoding task and memory type (*F* (2, 75) = 3.18, *p* = 0.05, *η*^*2*^ = 0.08). Further analysis showed that the recollection contributed more to gist memory after the naming task than after the description (*p* = 0.002) and the imagination (*p* < 0.001) tasks, while it contributed to detailed memory at a similar level after the naming and the description tasks (*p* = 0.34). The recollection contribution was the lowest after the imagination task (*p’s* < 0.001) (Fig 5C).

For the familiarity process, the results showed a significant main effect of memory type (detailed memory > gist memory) (*F* (1, 75) = 10.00, *p* = 0.002, *η*^*2*^ = 0.12) and a significant interaction between encoding task and memory type (*F* (2, 75) = 7.20, *p* = 0.001, *η*^*2*^ = 0.16). Further analysis indicated that the contribution of familiarity to gist memory was higher after the naming task than after the description task (*p* = 0.04), but comparable after the description task and the imagination task (*p* = 0.15). The contribution of familiarity to detailed memory was the highest after the description task (vs. naming, *p* = 0.05; vs. imagination, *p* = 0.007) (Fig 5D). The results suggest that the encoding tasks modulate the contributions of recollection and familiarity to different types of memories.

We also performed a separate ANOVA which included memory process (recollection, familiarity) as a factor. The results showed a significant interaction between memory process and memory type (*F* (1, 75) = 16.92, *p* = 0.001, *η*^*2*^ = 0.18). Further analysis showed that the recollection process contributed more than familiarity to both gist and detailed memories (*p* < 0.001). When the two memory types were compared, gist memory was more dependent on the recollection process (*p* = 0.003), whereas detailed memory showed the opposite (*p* = 0.002). There was also a significant interaction between memory process and retention interval (*F* (3, 225) = 60.92, *p* < 0.001, *η*^*2*^ = 0.45) (S2 Fig). Further analysis showed that memory processes changed over time for both gist and detailed memories. At shorter intervals, both memory types relied more on recollection (vs. familiarity) process (at 10 mins and 1 day, *p*’s < 0.001). But with the passage of time, the recollection contribution declined significantly, and the familiarity contribution decreased much less, which resulted in higher contribution of familiarity (vs. recollection) at longer intervals (at 1 month, *p* = 0.056).

Therefore, the results of the experimental comparison showed that gist memory and detailed memory had distinct accuracy and forgetting patterns after different encoding tasks. The accuracy of gist memory was the highest and its forgetting rate was the slowest after the naming task. The forgetting rate of detailed memory was the slowest after the description task. The naming task enhanced both the recollection and familiarity contributions, whereas the description task enhanced the familiarity contribution.

## Discussion

The objective of the present study was to explore to what extent the encoding task and recognition process modulated the forgetting of gist and detailed memories of visual objects. There were three main results. First, after the naming task, gist memory and detailed memory had similar forgetting rates, whereas after the description and the imagination tasks, gist memory was forgotten more rapidly than detailed memory. Second, when the performance after different tasks was compared, the forgetting rate of gist memory was the slowest after the naming task, while that of detailed memory was the slowest after the description task. Memory accuracy of gist and detailed information was also modulated by encoding task. Gist memory accurcy was the highest after the naming task, whereas detailed memory accuracy was higher after the description and the naming tasks than after the imagination task. Third, the naming task enhanced the contributions of recollection and familiarity to gist memory, while the description task mainly enhanced the contribution of familiarity to detailed memory. These results reveal the importance of the encoding task in the forgetting of gist and detailed information, and suggest a possible way to maintain perceptual details of objects at longer intervals.

### The forgetting of gist and detailed memories

One novelty of our results was that the forgetting rate of gist and detailed memories was modulated by the encoding tasks. Different aspects of an episode can be forgotten at different rates [58], but previous studies have had inconsistent findings on the forgetting rates of gist and detailed memories. Some studies found that detailed memory is forgotten more rapidly than gist memory [6, 26, 27], while others showed they have comparable forgetting rates [13, 30]. The discrepancies in forgetting may partly be due to the differences in encoding tasks. In this study, the naming task and the description task were used during the study phase in Experiments 1 and 2 separately. The naming task is commonly used for encoding pictures of common objects. In this task, the participants were required to verbally report the name of the pictures, so that the gist information of the object was emphasized. In contrast, in the description task, the participants were required to describe the object’s features, which emphasized the processing of perceptual details. The FTT proposes that the details and gist of an event are encoded in parallel and stored separately. Usually gist information is prioritized during encoding and retrieval [19–21]. Therefore, after the naming task, gist memory was higher than detailed memory at shorter intervals, which was similar to the results in previous studies with pictures [59] and film clips [6] as materials. However, the advantage for the lingering of gist information diminishes when the perceptual information is deliberately processed in our study in Experiment 2. Gist memory and detailed memory showed similar forgetting rates after the naming task, whereas the forgetting of detailed memory was significantly slower after the description than after the naming task. Similarly, when participants were instructed to study each image carefully in a repeated detection task and prepare for subsequent memory tests, which emphasized detailed information, Andermane and Bowers (2015) found that gist and detailed memories had similar forgetting rates [13].

In addition to the object naming and object description tasks, we adopted an imagination task to explore its effect on memory accuracy and forgetting of gist and detailed information. During this task, participants were asked to imagine a proper scene that was associated with the object. This task emphasized neither the gist nor the details of the objects. The results of Experiment 3 showed that after the imagination task, the performance of detailed memory was comparable to that of gist memory in general, but the forgetting of detailed memory was significantly slower than that of gist memory. It suggests that the imagination task could decrease the forgetting of perceptual features, although the detailed memory accuracy is not enhanced. The difference in memory forgetting was also manifested when the three tasks were compared. Gist memory was forgotten the slowest after the naming task, whereas detailed memory was forgotten the slowest after the description task. Taken together, these results suggest that encoding manipulation is an important factor to influence forgetting of gist and details, especially when the encoding task is explicitly oriented to a specific type of information (i.e., gist or details).

The results of different forgetting patterns were confirmed when initial memory performance was controlled and when a 1-month interval was included. The interaction between memory type and the retention interval is usually used in previous studies to determine the difference in memory forgetting [6, 13, 38, 49–51]. One problem with this approach, however, is that the initial performance of the gist and detailed memories may be different, which influences the interaction effect. For example, in the study of Sekeres et al. (2016), as participants recalled greater number of details (about 8) than gist (about 4) right after encoding, detailed memory had a larger space to decline over time, which led to its rapid forgetting [6]. In this study, we controlled for the initial performance and adopted different methods (i.e., forgetting rate calculation and forgetting curve fitting) [52, 53, 60] to clarify and illustrate the effect of encoding task on the forgetting of gist and details. The results confirmed that the encoding task modulated subsequent memory forgetting.

In addition to the forgetting analysis approach, we adopted an interval of 1-month in the memory tests. This could explain why we did not find rapid forgetting for detailed memory as previous studies have revealed [6, 59]. The time change of memory performance is usually observed within 1 week [6, 13, 59], which may not be sufficient to detect the pattern of forgetting at longer retention intervals. As shown in Experiment 1, we also found that gist memory performance was higher than detailed memory at 10 mins and 1 day. The results of the curve fitting showed that the difference between gist and detailed memories was mainly shown at intervals of 10 minutes or 1 day. However, when the retention interval was extended to 1 month, their forgetting pattern was similar [30]. In the description task, the difference between gist and detailed memories was mainly manifested at the intervals of 1 week and 1 month. Note that the findings of reaction times were in line with those of memory accuracy, i.e., higher accuracy was associated with quicker responses. These results suggest that memory may significantly change over the course of 1 week and beyond, so time intervals longer than a week are necessary to explore memory forgetting.

In this study, we used words and object pictures to test gist and detailed memories, respectively. Some may argue that the difference in the retrieval format of gist and detailed memories influences the forgetting patterns. For example, as the word format was used to test gist memory, which was different from the format during encoding, the participants may rely more on the recollection process during old/new word recognition. In addition, encoding specificity [61] was different in the three experiments, which may lead to the possibility that the word-retrieval cues are more effective than picture-retrieval cues after the naming task. Although we could not fully exclude this possibility, we believe that the different forgetting patterns in the experiments were not driven exclusively by encoding specificity, for the following reasons. First, the choice of different test formats was based on the definition of gist and detailed memory and the proposal that gist and detailed information could be encoded in parallel and retrieved independently [11, 18–21]. When the participants judged whether a concept word was old, they did not necessarily retrieve the picture information. Thus, it is reasonable that gist and detailed memories are tested in different formats [7, 11, 22]. Second, both conceptual and perceptual information could be processed during the three encoding tasks. Object naming is an automatic response when object images are presented, so even if participants were not asked to name the objects in the description and the imagination tasks, the conceptual representations could be processed automatically [11, 20, 21]. In the naming task, the result showed that even at 1-month interval, the detailed memory was above the chance level. It suggests that although the conceptual information of the objects has a priority to be processed, the perceptual information is not entirely ignored at encoding. In addition, the semantic or conceptual level of the objects was processed by the animate/inanimate judgment in three experiments. Therefore, the critical difference between the three tasks may be the degree of processing conceptual and perceptual information during encoding. Third, previous studies with pictures as the materials [13, 30, 59] obtained similar results. For example, when the level of similarity was used to distinguish gist and detailed memories, pictures were used as materials during both encoding and retrieval. The result showed that gist and detailed memories had comparable forgetting rates [13]. These findings indicate that different encoding tasks differently modulated the forgetting patterns of gist and details in three experiments.

### The mechanism of the forgetting—Recollection and familiarity processes

The current study also clarified the relationship between memory type and memory process and suggests a possible mechanism why different encoding tasks modulate the forgetting of gist and detailed memories. Andermane et al. (2021) proposed that the way in which information is originally encoded has a direct bearing on how it is forgotten [58]. Sadeh et al. (2014) also proposed that how we forget may depend on how we remember [40]. Memories depending on recollection are more vulnerable to decay than interference, and memories depending on familiarity show the opposite [34, 40]. Consistent with this proposal, the results showed a significant interaction between memory process and retention interval. It suggests that the contributions of recollection and familiarity change over time for both gist and detailed memories. At shorter intervals, both memory types relied more on recollection. But with the passage of time, the recollection contribution declined significantly, and the familiarity contribution declined to a lesser degree, which resulted in higher contribution of familiarity (vs. recollection) at longer intervals.

Furthermore, the results showed significant interactions between memory type and encoding task for both the recollection and familiarity processes. The encoding tasks modulated the contributions of recollection and familiarity to gist and detailed memories. Relative to the naming task, the description task enhanced the familiarity contribution to detailed memory. Because the familiarity process is more resistant to decay [40], when the task enhanced the familiarity contribution to detailed memory, its forgetting rate decreased. Similarly, relative to the description task, the naming task enhanced the familiarity contribution to gist memory, which may lead to a slower forgetting rate for gist memory. Therefore, our results suggest that the enhanced familiarity contribution is an important mechanism to decrease the forgetting rate. By this mechanism, encoding tasks could modulate the forgetting rates of gist and detailed memories. On the other hand, it is possible that the recollection contribution may also be associated with slower forgetting rate, as the naming task enhanced the recollection contribution to gist memory as well. But because the recollection significantly decreased over time, to what extent it contributes to memory forgetting needs further investigations.

The underlying mechanism for the imagination task may be more complex than that for the naming and the description tasks. Previous studies indicated that encoding pictures with scenes facilitated memory for object names, but not for object details [43]. Our study also showed lower detailed (vs. gist) memory at 10 minutes in Experiment 3. On the other hand, although the detailed memory was not enhanced at the shorter interval, it was forgotten more slowly than gist memory over time. The results of Experiment 3 showed that the familiarity contribution was higher for detailed (vs. gist) memory from 1 day to 1 month. In addition, when the three tasks were compared, the recollection contribution was the lowest for both gist and detailed memories after the imagination task. Therefore, it is possible that imagining a scene that was associated with an object induces unitized object representations [62, 63]. The unitized representations increases the contribution of familiarity to long-term memory and benefits the retention of perceptual features. Imagination tasks are often used as memory encoding tasks, and studies have proved that imagination is a good way to generate unitized representations [64, 65]. In these studies, participants are asked to create an image/scenario for an object, or imagine an object/word-denoted object in an associated color [62, 66, 67]. In addition, when two separate items or features are unitized, the associated memory could be enhanced by the familiarity contribution. For example, in a study of Rhodes and Donaldson (2008), after participants encoded the unrelated word pairs by interactive imagery (i.e., create an image of the two items interacting together), the familiarity contribution was enhanced and memory for the word pairs was improved [64]. As memory dependent on the familiarity process is more resistant to decay, detailed memory was forgotten less slowly than gist memory in Experiment 3.

The RKG procedure is widely used to estimate the underlying processes during recognition based on the dual-process model [34, 48, 68]. When this procedure is used, it is important to ensure that participants have a proper appreciation of the distinction between remember, know and guess responses. In this study, our instructions and analyses were strictly based on the literature of RKG [33, 34, 48]. In addition, to ensure that they understood and followed the RKG instructions, the participants were asked to practise doing the RKG responses with feedback from experimenters before the formal test. The distinction in recollection and familiarity is thus suitable to explain the current findings on memory and forgetting.

### The accuracy of gist and details

In addition to the findings of forgetting of gist and detailed information, the results showed that the two types of memory were modulated by the encoding task [12, 69]. The accuracy of gist memory was the highest after the naming task, while the accuracy of detailed memory was higher after the description and the naming (vs. imagination) tasks. When the participants were oriented to process a specific type of information, the corresponding type of memory was improved. When neither the gist nor the detailed aspect was emphasized in the imagination task, memory performance was the lowest and the forgetting was the fastest for both gist and detailed memories.

The higher gist memory after the naming (vs. description and imagination) task was consistent with our hypothesis. Based on the FTT, gist and verbatim traces of one experience are stored in parallel and retrieved separately. Thus, both perceptual and conceptual representations can coexist and these can be independently manipulated. The parallel representations make it possible for us to use different encoding tasks to selectively enhance gist and detailed memory traces [7, 11, 22]. Specifically, relative to the description and imagination task, naming the objects offered a verbal coding for the concepts, which corresponds to the verbal/conceptual representation of gist information and leads to improved gist memory.

By describing the details of the objects, the perceptual representation was greatly processed, which could lead to improved detailed memory. This hypothesis was supported by the better detailed memory after the description than after the imagination task, but was not illustrated when the description task and the naming task was compared. This could be explained by the following reasons. First, the perceptual information could also be processed after the naming task, as the results showed that even at 1-month interval, the detailed memory was above chance after the naming task. This may lead to a non-significant difference in detailed memory between the description and the naming task. Second, the description task may not be sufficient to enhance recollection-based memory. The study of McCrudden (2019) also found similar results, with no significant improvement in detailed memory. In the study of McCrudden (2019), pre-reading questions targeted to the main ideas or detailed text segments were applied to improve gist or detailed memory separately [12]. The results showed that gist memory was enhanced when main idea of the texts was attended, but detailed memory was not significantly different after the two encoding conditions [12]. As shown in our results, the level of detail rated in the description task was moderate, so the description manipulation may not improve the accuracy of detailed memory by recollection. This explanation is also supported by the results of higher familiarity contribution, rather than recollection, for detailed memory in three experiments. It is possible that only when detailed information associated with object/event contexts is strongly emphasized, could the detailed memory be significantly enhanced. For example, in a study of Grilli et al. (2019), an episodic specificity induction (ESI) was applied, by which participants recalled or imagined details of an autobiographical memory and changed their mode of thinking to an episodic one [41]. The results showed that recall of perceptual details of film clips was significantly improved after the ESI than after the gist-based induction. Thus, describing pictures may not require retrieving episodic details from past experiences and thus could enhance non-recollected processes [42, 70]. We should also consider other features, such as typicality (i.e., the congruence with prior knowledge) [71, 72] and level of entity (the integration of perceptual features and conceptual features of an object) [73, 74], that may help to clarify the mechanisms of familiarity-based detailed memory.

It is a bit counterintuitive that detailed memory relied more on the familiarity process, rather than the recollection process. The interaction between memory type and memory process was significant, which indicated that gist memory depended more on the recollection process, while detailed memory depended more on the familiarity process. But note that when the contributions of recollection and familiarity were directly compared, the results showed that both gist and detailed memories relied more on the recollection than the familiarity process, which is consistent with previous findings on memory recognition [32, 55, 75]. Some researchers suggested that both memory processes and representation contents should be taken into consideration [76]. Similarly, we consider that this finding is associated with object entity. Different from other materials, object gist and details are remembered at high accuracy when thousands of images are viewed only once, and the familiarity process contributes to detailed memory of objects [15, 29, 77]. Neuroimaging studies have suggested that object entity representation increases the activation of the perirhinal cortex and the hippocampus, whereas the contextual or associative information depends on the hippocampus and cortical regions [66, 78–80]. The perirhinal cortex is important for object encoding, consolidation and retrieval [58, 81–83], and the associated memory is familiarity-based [32, 82, 84]. In contrast, the hippocampus is involved in when detailed or contextual information is retained [85]. Different from contextual details such as temporal or spatial information of an object, the features like color and shape are represented as an entity, and this process is familiarity-based and may depend on the perirhinal cortex.

### The limitations and future directions

Our study has some limitations for future investigations. First, gist and detail memories were tested by different forms of materials and instructions. Although the results showed that encoding tasks modulated subsequent memory performance of gist and details, the gist and detailed memories differed in the way they were measured. In addition, the three encoding tasks may result in a potential issue of encoding-retrieval specificity [61]. For example, the naming task may have made word-retrieval cues more effective than picture image cues. It is important to point out that memory performance could also be influenced by the match between encoding and retrieval, i.e., memory differs how the encoding specificity is manipulated. Further studies are needed to explore this issue when the same task formats are used and when another condition with the written words is added during encoding.

Second, whether the results could be generalized to other materials needs further investigation. Particularly, the detailed memory of objects relied more on the familiarity, which is the main reason why it was forgotten more slowly at longer intervals in this study. But for words and scenes, the detailed memory seems to rely more on the recollection process [32]. As both memory content and memory process should be taken into consideration when memory change is studies [76], it is necessary to clarify to what extent memory contents interact with memory process to influence forgetting.

Third, in this study, the encoding task was treated as a between-subjects factor, while memory type and retention interval were as within-subjects factors. The within-subjects factor could diminish individual difference in memory performance for the effect of that particular factor. For example, if four groups of participants were tested at only one retention interval (i.e., the factor of retention interval was treated as a between-subjects factor), the memory difference across time may be confounded by individual difference between the groups. But at the same time, we could not examine whether the information forgotten at previous memory test would be reinstated spontaneously at later retention intervals, and whether individual difference of naming, description and imagination abilities of three groups of participants would influence subsequent memory and forgetting. Future studies could change the within- and between-subjects factors to explore these interesting issues.

Fourth, in Experiment 3, we only asked the participants to rate the vividness, and did not ask them to report what they had imagined. As the imagination instruction was not specific, participants may adopt various strategies to perform this task. They may imagine a scene that was associated with an object and its perceptual features, or they may imagine an episode that combines a scene and an object’s entity. We are also unsure whether the imagined scenes were the same for different objects and to what extent the scenes were compatible with the objects. The results showed that the correlation between vividness and detailed memory accuracy was not significant. Future studies could ask participants to describe specific contents of the scenes they imagined (e.g., how unusual the imagination is, whether individuals imagine a story or a vivid visual scene) to clarify the relationship between imagination and memory performance of different aspects.

## Conclusion

Our study found that the encoding task modulated memory performance and forgetting of gist and detailed information. After the naming task, participants forgot gist and detailed memories at similar rates, but after the description and the imagination tasks, their detailed memory was maintained for a long time with a slower decline. The forgetting rate of gist memory was the slowest after the naming task, whereas that of detailed memory was the slowest after the description task. By using description encoding strategies, the familiarity process contributed more to detailed memory, which made detailed memory less susceptible to decay over time. The results provide a possible way to maintain detailed memory for a longer time by the detailed description of object images in healthy people and patients with amnesia.

## Supporting information

S1 Fig The results of RTs in each experiment. (A). RTs for corrected gist and detailed memory in Experiment 1. (B). RTs for corrected gist and detailed memory in Experiment 2. (C). RTs for corrected gist and detailed memory in Experiment 3. The error bars represent the standard errors of the means. * *p* < 0.05; ** *p* < 0.01; *** *p* < 0.001.
(TIF)

S2 Fig The interaction of memory process * retention interval. Over 1 month, the contribution of recollection decays faster than the contribution of familiarity.
(TIF)

S1 Table The results of d’, hit rate (Hit) and false alarm rate (FA). (DOCX)

S2 Table The P value, 95% CIs of the pairwise comparisons for retention interval. (DOCX)

S3 Table The P value, 95% CIs of the pairwise comparisons for memory type * retention interval. (DOCX)
