# Supplementary figures and images for: The effect of encoding task on the forgetting of object gist and details
Source: PLoS One. 2021 Sep 22;16(9):e0255474. doi: 10.1371/journal.pone.0255474 (PMC8457468; doi:10.1371/journal.pone.0255474)

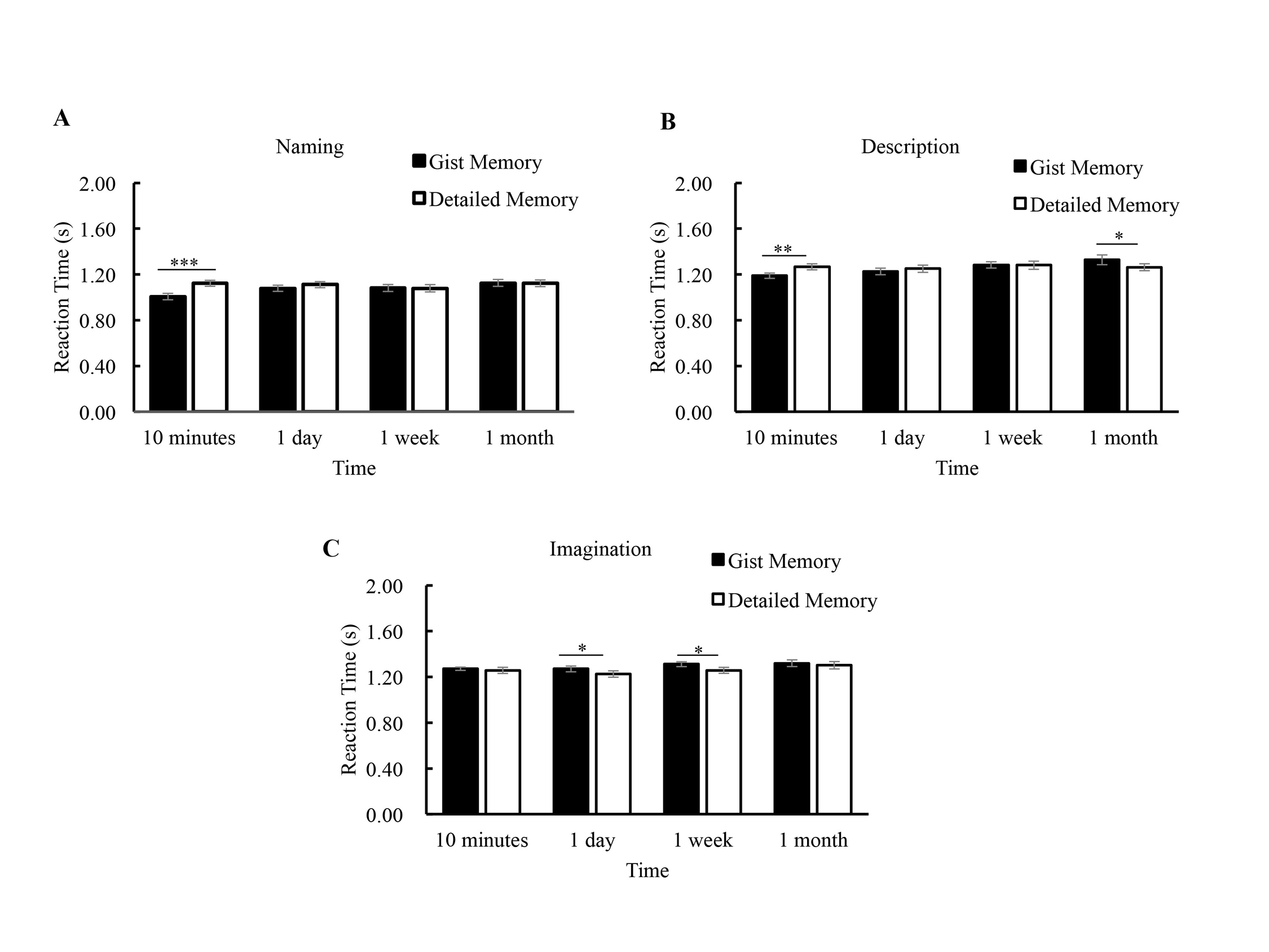

Supplement: S1 Fig — (A). RTs for corrected gist and detailed memory in Experiment 1. (B). RTs for corrected gist and detailed memory in Experiment 2. (C). RTs for corrected gist and detailed memory in Experiment 3. The error bars represent the standard errors of the means. * p < 0.05; ** p < 0.01; *** p < 0.001. (TIF) [file pone.0255474.s001.tif]

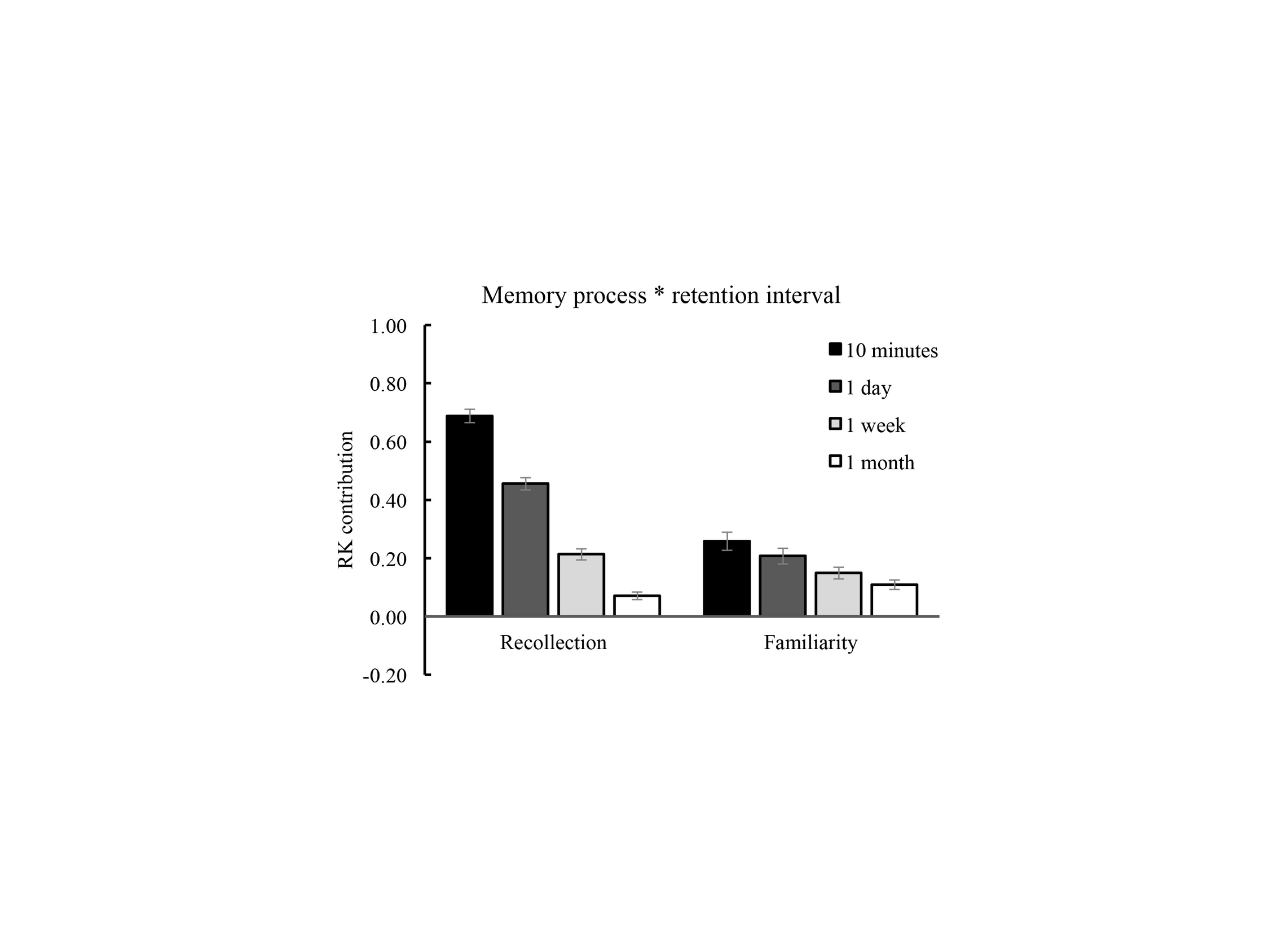

Supplement: S2 Fig — Over 1 month, the contribution of recollection decays faster than the contribution of familiarity. (TIF) [file pone.0255474.s002.tif]
