# Supplementary material for: The effect of encoding task on the forgetting of object gist and details
Source: PLoS One. 2021 Sep 22;16(9):e0255474. doi: 10.1371/journal.pone.0255474 (PMC8457468; doi:10.1371/journal.pone.0255474)
Supplement: S1 Table — (DOCX) [file pone.0255474.s003.docx]

**S1 Table.** **The Results of d’, hit rate (Hit) and false alarm rate (FA)**

|  |  | 10 minutes | | 1 day | | 1 week | | 1 month | |  |
| --- | --- | --- | --- | --- | --- | --- | --- | --- | --- | --- |
|  |  | Gist memory | Detailed memory | Gist memory | Detailed memory | Gist memory | Detailed memory | Gist memory | Detailed memory | |
|  |  |  |  |  |  |  |  |  |  |  |
| Naming (EXP 1) | d’  (SD) | 2.90 (0.72) | 2.19 (0.54) | 1.96 (0.65) | 1.40 (0.55) | 0.99 (0.56) | 0.90 (0.53) | 0.56 (0.57) | 0.58 (0.56) | |
|  | hit  (SD) | 0.94 (0.07) | 0.91 (0.08) | 0.81 (0.11) | 0.83 (0.09) | 0.64 (0.16) | 0.64 (0.16) | 0.59 (0.18) | 0.49 (0.17) | |
|  | FA  (SD) | 0.11 (0.14) | 0.24 (0.17) | 0.18 (0.12) | 0.36 (0.17) | 0.30 (0.15) | 0.33 (0.17) | 0.39 (0.17) | 0.29 (0.15) | |
| Description (EXP 2) | d’  (SD) | 2.27 (0.69) | 2.05 (0.66) | 1.29 (0.67) | 1.42 (0.70) | 0.56 (0.60) | 1.13 (0.62) | 0.23 (0.50) | 0.92 (0.51) | |
|  | hit  (SD) | 0.87 (0.09) | 0.87 (0.09) | 0.72 (0.14) | 0.81 (0.12) | 0.57 (0.17) | 0.72 (0.16) | 0.51 (0.17) | 0.58 (0.13) | |
|  | FA  (SD) | 0.17 (0.15) | 0.22 (0.15) | 0.29 (0.17) | 0.34 (0.15) | 0.38 (0.16) | 0.33 (0.16) | 0.42 (0.15) | 0.26 (0.13) | |
| Imagination (EXP 3) | d’  (SD) | 2.15 (0.71) | 1.71 (0.72) | 1.11 (0.58) | 1.09 (0.61) | 0.33 (0.70) | 0.69 (0.60) | 0.08 (0.55) | 0.54 (0.53) | |
|  | hit  (SD) | 0.86 (0.11) | 0.82 (0.14) | 0.69 (0.15) | 0.71 (0.15) | 0.52 (0.22) | 0.56 (0.15) | 0.47 (0.14) | 0.47 (0.15) | |
|  | FA  (SD) | 0.18 (0.12) | 0.27 (0.14) | 0.31 (0.17) | 0.34 (0.17) | 0.41 (0.18) | 0.32 (0.16) | 0.44 (0.17) | 0.29 (0.17) | |
