# Supplementary material for: The effect of encoding task on the forgetting of object gist and details
Source: PLoS One. 2021 Sep 22;16(9):e0255474. doi: 10.1371/journal.pone.0255474 (PMC8457468; doi:10.1371/journal.pone.0255474)
Supplement: S2 Table — (DOCX) [file pone.0255474.s004.docx]

**S2 Table.** **The P value, 95% CIs of the pairwise comparisons for retention interval**

|  |  | Naming | | Description | | Imagination | |
| --- | --- | --- | --- | --- | --- | --- | --- |
|  |  | p value | 95% CI | p value | 95% CI | p value | 95% CI |
| Corrected  recognition | 10 min vs. 1 day | < 0.001 | [0.12, 0.28] | < 0.001 | [0.13, 0.31] | < 0.001 | [0.14, 0.34] |
|  | 10 min vs. 1 week | < 0.001 | [0.32, 0.53] | < 0.001 | [0.30, 0.47] | < 0.001 | [0.34, 0.54] |
|  | 10 min vs. 1 month | < 0.001 | [0.47, 0.64] | < 0.001 | [0.39, 0.55] | < 0.001 | [0.39, 0.63] |
|  | 1 day vs. 1 week | < 0.001 | [0.12, 0.33] | < 0.001 | [0.08, 0.25] | < 0.001 | [0.09, 0.31] |
|  | 1 day vs. 1 month | < 0.001 | [0.26, 0.45] | < 0.001 | [0.16, 0.33] | < 0.001 | [0.20, 0.35] |
|  | 1 week vs. 1month | = 0.010 | [0.02, 0.24] | = 0.030 | [0.01, 0.17] | = 0.489 | [-0.04, 0.19] |
| Reaction  Time | 10 min vs. 1 day | = 0.569 | [-0.08, 0.02] | = 1.000 | [-0.09, 0.07] | = 1.000 | [-0.04, 0.07] |
|  | 10 min vs. 1 week | = 1.000 | [-0.09, 0.05] | = 0.805 | [-0.15, 0.05] | = 1.000 | [-0.07, 0.03] |
|  | 10 min vs. 1 month | = 0.321 | [-0.14, 0.02] | = 0.594 | [-0.18, 0.05] | = 0.802 | [-0.13, 0.04] |
|  | 1 day vs. 1 week | = 1.000 | [-0.04, 0.07] | = 0.696 | [-0.12, 0.03] | = 0.716 | [-0.10, 0.03] |
|  | 1 day vs. 1 month | = 1.000 | [-0.11, 0.05] | = 0.535 | [-0.15, 0.04] | = 0.528 | [-0.17, 0.04] |
|  | 1 week vs. 1month | = 0.332 | [-0.10, 0.02] | = 1.000 | [-0.08, 0.06] | = 1.000 | [-0.11, 0.06] |
| Recollection | 10 min vs. 1 day | < 0.001 | [0.15, 0.32] | < 0.001 | [0.13, 0.33] | < 0.001 | [0.16, 0.36] |
|  | 10 min vs. 1 week | < 0.001 | [0.35, 0.59] | < 0.001 | [0.39, 0.58] | < 0.001 | [0.32, 0.56] |
|  | 10 min vs. 1 month | < 0.001 | [0.59, 0.76] | < 0.001 | [0.46, 0.66] | < 0.001 | [0.39, 0.61] |
|  | 1 day vs. 1 week | < 0.001 | [0.14, 0.33] | < 0.001 | [0.17, 0.33] | < 0.001 | [0.08, 0.29] |
|  | 1 day vs. 1 month | < 0.001 | [0.37, 0.51] | < 0.001 | [0.25, 0.42] | < 0.001 | [0.16, 0.33] |
|  | 1 week vs. 1month | < 0.001 | [0.11, 0.30] | = 0.003 | [0.02, 0.14] | = 0.149 | [-0.01, 0.13] |
| Familiarity | 10 min vs. 1 day | = 0.389 | [-0.04, 0.20] | = 1.000 | [-0.12. 0.16] | = 0.335 | [-0.04, 0.21] |
|  | 10 min vs. 1 week | = 0.001 | [0.06, 0.29] | = 1.000 | [-0.14, 0.22] | = 0.004 | [0.05, 0.32] |
|  | 10 min vs. 1 month | = 0.001 | [0.07, 0.31] | = 0.346 | [-0.05, 0.26] | < 0.001 | [0.09, 0.37] |
|  | 1 day vs. 1 week | = 0.075 | [-0.01, 0.20] | = 1.000 | [-0.11, 0.15] | = 0.050 | [0.00, 0.19] |
|  | 1 day vs. 1 month | = 0.124 | [-0.02, 0.24] | = 0.424 | [-0.04, 0.21] | < 0.001 | [0.06, 0.23] |
|  | 1 week vs. 1month | = 1.000 | [-0.08, 0.11] | = 0.415 | [-0.03, 0.16] | = 1.000 | [-0.05, 0.15] |
