# Supplementary material for: The effect of encoding task on the forgetting of object gist and details
Source: PLoS One. 2021 Sep 22;16(9):e0255474. doi: 10.1371/journal.pone.0255474 (PMC8457468; doi:10.1371/journal.pone.0255474)
Supplement: S3 Table — (DOCX) [file pone.0255474.s005.docx]

**S3 Table.** **The P value, 95% CIs of the pairwise comparisons for memory type * retention interval**

|  |  |  | Naming | | Description | | Imagination | |  |
| --- | --- | --- | --- | --- | --- | --- | --- | --- | --- |
|  |  |  | P value | 95% CI | P value | 95% CI | P value | 95% CI |  |
| Corrected  recognition | GM vs. DM | 10 minutes | < 0.001 | [0.10, 0.24] | = 0.188 | [-0.03, 0.14] | = 0.007 | [0.04, 0.21] |  |
|  |  | 1 day | = 0.001 | [0.08, 0.26] | = 0.410 | [-0.13, 0.06] | = 0.960 | [-0.09, 0.10] |  |
|  |  | 1 week | = 0.385 | [-0.04, 0.11] | = 0.001 | [-0.29, -0.09] | = 0.029 | [-0.25, -0.01] |  |
|  |  | 1 month | = 0.939 | [-0.09, 0.10] | < 0.001 | [-0.34, -0.14] | = 0.011 | [-0.25, -0.04] |  |
| Reaction  Time | GM vs. DM | 10 minutes | < 0.001 | [-0.18, -0.06] | = 0.005 | [-0.13, -0.03] | = 0.524 | [-0.03, 0.06] |  |
|  |  | 1 day | = 0.214 | [-0.08, 0.02] | = 0.453 | [-0.09, 0.04] | = 0.045 | [0.00, 0.09] |  |
|  |  | 1 week | = 0.878 | [-0.05, 0.06] | = 0.906 | [-0.05, 0.05] | = 0.015 | [0.01, 0.10] |  |
|  |  | 1 month | = 0.926 | [-0.05, 0.05] | = 0.028 | [0.01, 0.12] | = 0.492 | [-0.03, 0.07] |  |
| Recollection | GM vs. DM | 10 minutes | < 0.001 | [0.11, 0.23] | = 0.038 | [0.01, 0.20] | = 0.007 | [0.05, 0.26] |  |
|  |  | 1 day | < 0.001 | [0.06, 0.19] | = 0.851 | [-0.12, 0.10] | = 0.101 | [-0.02, 0.16] |  |
|  |  | 1 week | = 0.668 | [-0.06, 0.10] | = 0.349 | [-0.14, 0.05] | = 0.441 | [-0.09, 0.04] |  |
|  |  | 1 month | = 0.348 | [-0.05, 0.12] | = 0.140 | [-0.11, 0.02] | = 0.416 | [-0.09, 0.04] |  |
| Familiarity | GM vs. DM | 10 minutes | = 0.763 | [-0.26, 0.20] | = 0.359 | [-0.18, 0.07] | = 0.241 | [-0.06, 0.21] |  |
|  |  | 1 day | = 0.636 | [-0.10, 0.16] | = 0.001 | [-0.36, -0.11] | = 0.054 | [-0.16, 0.00] |  |
|  |  | 1 week | = 0.366 | [-0.04, 0.11] | < 0.001 | [-0.28, -0.12] | = 0.226 | [-0.12, 0.03] |  |
|  |  | 1 month | = 0.970 | [-0.08, 0.08] | = 0.029 | [-0.04, -0.01] | = 0.042 | [-0.11, 0.00] |  |
| * GM: gist memory, DM: detailed memory | | | | | | | | | |
